# Digital Health Technology for Improving Physical Function in Adults With Chronic Heart Failure: Systematic Review and Meta-Analysis of Randomized Controlled Trials

**DOI:** 10.2196/91820

**Published:** 2026-08-13

**Authors:** Zhe Meng, Juncai Li, Jing Yang, Yijia Lin, Qirui Zhang, Longjie Wei, Xiuling Zhou

**Affiliations:** 1 School of Nursing, Changchun University of Chinese Medicine, 1035 Boshuo Road, Jingyue Economic and Technological Development Zone, ChangChun, Jilin, 130117, China; 2 The Affiliated Hospital of Changchun University of Chinese Medicine, Nanguan District, Changchun, Jilin, 130117, China, 86 15948000732

**Keywords:** heart failure, digital health, cardiac rehabilitation, telemedicine, exercise tolerance, meta-analysis

## Abstract

**Background:**

Chronic heart failure (CHF) significantly impairs physical function and quality of life. Although exercise-based cardiac rehabilitation represents a primary therapeutic strategy, participation rates remain low due to logistical barriers. Digital health technologies (DHTs) offer a promising alternative to deliver home-based interventions. However, evidence regarding their specific impact on functional capacity versus daily physical behavior remains inconsistent.

**Objective:**

This systematic review and meta-analysis aimed to evaluate the efficacy of DHTs on physical activity and health outcomes in patients with CHF and to identify the intervention characteristics that drive physiological improvement.

**Methods:**

We searched Embase, CENTRAL, PubMed, Web of Science, and Joanna Briggs Institute Evidence-Based Practice (JBI EBP) database for randomized controlled trials published between August 2015 and July 2025. Studies comparing DHT interventions with usual care or nondigital active controls in adults with CHF were included. Primary outcomes were functional exercise capacity (6-minute walk distance), cardiorespiratory fitness (peak oxygen uptake), and daily step count. Secondary outcomes included health-related quality of life (Minnesota Living with Heart Failure Questionnaire and Kansas City Cardiomyopathy Questionnaire). Data were synthesized using random-effects models, and the certainty of evidence was assessed using the GRADE (Grading of Recommendations Assessment, Development, and Evaluation) approach.

**Results:**

In total, 16 unique randomized controlled trials (across 20 publications) involving 3441 participants were included. DHT interventions significantly improved 6-minute walk distance (mean difference [MD] 18.74 m, 95% CI 9.31‐28.17; *P*<.001) and peak oxygen uptake (MD 0.84 mL/kg/minute, 95% CI 0.59‐1.09; *P*<.001) compared with controls. Subgroup analysis revealed that comprehensive hybrid telerehabilitation models drove consistent physiological benefits, whereas standalone app-based interventions demonstrated a uniformly nonsignificant effect (*I*^2^=0%). DHTs also significantly reduced symptom-related quality of life burden (Minnesota Living with Heart Failure Questionnaire: MD –5.63, 95% CI –8.55 to –2.71; *P*<.001), but narrative synthesis revealed highly inconsistent effects on daily step counts, and pooled analysis showed no significant impact on general health status (Kansas City Cardiomyopathy Questionnaire: *P*=.95). No significant increase in adverse events was reported.

**Conclusions:**

DHTs are effective strategies to improve cardiorespiratory fitness and functional capacity in patients with CHF. However, physiological gains do not automatically guarantee increased daily physical activity. The safety and efficacy of these interventions appear to depend on the delivery mode, with structured hybrid models incorporating remote supervision outperforming passive app-based approaches. Future implementation should prioritize personalized, medically supervised closed-loop management systems.

## Introduction

Chronic heart failure (CHF) remains a prevalent and burdensome global health challenge, affecting over 64 million individuals worldwide [1,2]. While survival rates have improved due to advances in pharmacological and device-based therapies [3], patients with CHF continue to experience severe exercise intolerance and physical deconditioning [4-7]. This functional decline is not merely a symptom; reduced exercise capacity, often quantified by peak oxygen uptake (peak VO_2_) or the 6-minute walk distance (6MWD), is a potent independent predictor of rehospitalization and mortality [8,9]. Consequently, international guidelines prioritize improving physical function and maintaining an active lifestyle as critical strategies to enhance prognosis and quality of life.

Exercise-based cardiac rehabilitation represents a primary therapeutic strategy to reverse physical deconditioning and improve clinical outcomes. However, the delivery of conventional center-based cardiac rehabilitation faces substantial logistical barriers, including transportation difficulties, high costs, and limited facility capacity, resulting in persistently low participation rates of 10%‐30% [10-12]. Although the COVID-19 pandemic catalyzed the adoption of remote care models, the necessity for home-based alternatives extends beyond the crisis. Enduring barriers such as geographical inaccessibility and patient frailty continue to drive the demand for flexible, scalable rehabilitation solutions.

Digital health technologies (DHTs), ranging from smartphone apps and wearable sensors to comprehensive telerehabilitation platforms, have emerged as a promising solution to bridge this care gap [13]. Advancing beyond traditional telephone-based support, modern DHTs leverage real-time physiological monitoring, automated feedback algorithms, and interactive gamification to deliver personalized interventions directly to patients’ homes. These technologies aim to enhance self-efficacy and overcome barriers to exercise by providing continuous supervision and behavioral nudges outside the clinical setting [14-16].

Despite theoretical advantages, evidence regarding the efficacy of DHTs on physical outcomes remains inconsistent. While some systematic reviews have supported the benefits of telerehabilitation, they predominantly focused on “hard” end points like mortality or hospitalization, often overlooking granular physical activity behaviors such as daily step counts [17,18]. Furthermore, results from recent randomized controlled trials (RCTs) have been conflicting: some comprehensive hybrid models reported robust gains in exercise capacity, whereas trials using standalone mobile apps or passive monitoring often failed to demonstrate superiority over usual care. Crucially, existing reviews often conflated these diverse interventions by grouping simple SMS text messaging reminders with complex, medically supervised programs, thereby obscuring which specific types of DHT were truly effective. Additionally, the rapid evolution of technology means that older reviews including legacy technologies (eg, landline-based support) may not reflect the efficacy of modern smartphone-based and wearable-integrated interventions.

Therefore, this systematic review and meta-analysis focused on RCTs published in the last decade to synthesize contemporary evidence. Beyond evaluating standard clinical outcomes, we specifically aimed to determine whether digital interventions exert differential effects on functional capacity versus daily physical behavior. Furthermore, we conducted subgroup analyses to investigate whether efficacy is modified by the intervention modality, specifically distinguishing between comprehensive hybrid models (featuring closed-loop clinical supervision) and standalone app-based counseling. This approach aims to provide precise, actionable evidence to guide future digital health design and clinical implementation.

## Methods

### Overview

This systematic review and meta-analysis was conducted in accordance with the Cochrane Handbook for Systematic Reviews of Interventions and the PRISMA (Preferred Reporting Items for Systematic Reviews and Meta-Analyses) 2020 guidelines. The completed PRISMA checklist is provided in Checklist 1. The study protocol was prospectively registered in the International Prospective Register of Systematic Reviews (PROSPERO; registration CRD420251252258). The final methods, including all evaluated outcomes and preplanned subgroup analyses, were conducted in strict alignment with this registered protocol. As this study used exclusively published data, institutional review board approval was not required.

### Search Strategy

A comprehensive literature search was performed to capture contemporary technological interventions published between August 2015 and July 2025. We searched Embase, the Cochrane CENTRAL, the Joanna Briggs Institute Evidence-Based Practice (JBI EBP) database, PubMed, and Web of Science. The search strategies were developed based on MeSH, Emtree terms, and free-text terms in titles and abstracts (Multimedia Appendix 1). Key search terms included digital health, digital technology, internet-based intervention, telemedicine, virtual reality, wearable electronic devices, artificial intelligence, and heart failure. Reference lists of included papers and relevant reviews were also screened to identify additional eligible studies.

### Eligibility Criteria

#### Participants

We included studies involving adults (≥18 years) with a confirmed diagnosis of CHF, regardless of ejection fraction or New York Heart Association functional class (I-IV). Studies recruiting mixed populations were considered eligible if ≥80% of participants had CHF or if data regarding the CHF subgroup could be disaggregated. We excluded studies targeting patients with acute decompensated heart failure, those with heart transplants or left ventricular assist devices, and individuals with congenital heart disease.

#### Intervention

We defined DHT as any health intervention primarily delivered through digital or mobile platforms. Eligible interventions met one or more of the following criteria: (1) telemonitoring: automated transmission of physiological or behavioral data via digital devices, with or without feedback to users or clinicians; (2) app- or web-based interventions: structured programs delivered through mobile apps or web platforms, encompassing exercise prescription, self-care education, or behavior change support; (3) wearable device interventions: sensors or devices (eg, smartwatches and fitness trackers) collecting health-related data or providing real-time feedback; and (4) digital communication tools: remote interactions via SMS text messaging, push notifications, videoconferencing, or app-based messaging aimed at education, motivation, or clinical guidance.

We excluded interventions relying solely on nondigital methods (eg, paper-based diaries and standard landline telephone support without data transmission) or those where digital components were merely incidental (eg, administrative appointment reminders).

#### Comparison

Eligible comparators included usual care, enhanced usual care (eg, nondigital education or standard telephone follow-up), or active nondigital interventions. We excluded studies where the control group received a competing digital intervention.

#### Outcomes

To be included, studies had to report on at least 1 primary outcome pertaining to physical function or behavior. Studies reporting only secondary outcomes were excluded.

We focused on objectively measured functional and behavioral end points: (1) functional exercise capacity: measured by the 6MWD in meters; (2) cardiorespiratory fitness: measured by peak VO_2_ in mL/kg/minute derived from cardiopulmonary exercise testing; and (3) physical activity behavior: objectively measured daily step counts or minutes of moderate-to-vigorous physical activity assessed via accelerometers or pedometers (self-reported activity logs were excluded from this category).

Secondary outcomes included (1) disease-specific health status: scores from validated questionnaires, specifically the Kansas City Cardiomyopathy Questionnaire (KCCQ; including Overall Summary and Physical Limitation scores) or the Minnesota Living with Heart Failure Questionnaire (MLHFQ); and (2) general quality of life: evaluated using generic tools (eg, 36-Item Short Form Health Survey SF-36 and EQ-5D). We excluded outcomes measured using nonvalidated tools or those reported without predefined measurement protocols.

### Study Design

Eligible studies were restricted to RCTs published in English with full-text availability. We limited inclusion to studies published from August 2015 to July 2025. This time frame was selected to ensure that the findings reflected modern DHTs (eg, smartphones and wearables) rather than legacy systems (eg, landline-based telemonitoring), thereby ensuring high applicability to current clinical practice. We excluded observational studies, study protocols, conference abstracts, and non–peer-reviewed publications.

### Study Selection and Data Extraction

All search results were imported into EndNote (version 21; Clarivate Analytics), and duplicate records were removed. Two reviewers (ZM and JL) independently screened titles, abstracts, and full texts to assess eligibility based on the predefined criteria. Data extraction was performed independently by the same reviewers using a standardized, pilot-tested data extraction form. Extracted information included the first author, year of publication, country, type of DHT, type of heart failure, sample size, intervention and control group details, intervention duration, follow-up period, outcome measures (mean values and SDs), and reported clinical outcomes. We attempted to contact corresponding authors to obtain missing data where necessary. Any disagreements during study selection or data extraction were resolved through discussion with a third reviewer (XZ). Furthermore, we extracted methodological data regarding the reporting of safety outcomes, specifically noting how adverse events (AEs) were defined, which trials reported them, and whether the AE data collection mechanism was active or systematic (eg, using predefined checklists or clinical audits) versus passive (eg, relying on spontaneous patient self-reporting).

### Assessment of Methodological Quality

Two independent reviewers (ZM and JL) evaluated the risk of bias for each included study using the Cochrane Risk of Bias tool version 2. We assessed the risk of bias across 5 key domains: randomization process, deviations from intended interventions, missing outcome data, measurement of the outcome, and selection of the reported result. Specifically, we assessed the effect of assignment to intervention (the intention-to-treat principle). Visual representations of the risk of bias assessments were generated using the tool. Disagreements between reviewers were resolved through discussion with a third reviewer (XZ).

### Data Analysis and Synthesis

Statistical analyses were performed using Review Manager (version 5.4.1; The Cochrane Collaboration). To strictly prevent unit-of-analysis errors and double-counting, we meticulously screened for multiple publications originating from the same primary RCT (eg, subanalyses based on etiology or specific comorbidities). In all quantitative syntheses, each trial was included only once per outcome, using the data from the primary overall cohort, while secondary subcohorts sharing the same control group were excluded from the pooled meta-analysis in accordance with Cochrane guidelines. Quantitative synthesis was conducted when 3 or more studies reported the same outcome; otherwise, a narrative synthesis was adopted [19]. Continuous outcomes were summarized as mean differences (MDs) or standardized mean differences with 95% CIs, depending on whether the measurement scales were consistent across studies [20]. Heterogeneity was assessed via the Cochran *Q* test and the *I*^2^ statistic. Given the anticipated clinical and methodological heterogeneity inherent in digital health interventions, we applied a random-effects model for all primary analyses to provide a conservative estimate of treatment effects [21]. Subgroup and sensitivity analyses were also performed to investigate potential sources of heterogeneity and the stability of the results. To ensure transparency and reproducibility in these subgroup analyses, we established strict operational definitions for the intervention taxonomy. The presence or absence of specific “active ingredients” (eg, structured exercise prescription, human-in-the-loop remote supervision, and real-time physiologic monitoring) was mapped for each trial in an intervention component matrix (Multimedia Appendix 2) [22-41]. Based on this matrix, interventions were classified into two subgroups: (1) comprehensive or hybrid telerehabilitation, defined as programs simultaneously incorporating a structured exercise prescription, human-in-the-loop remote supervision, and continuous closed-loop physiological or symptom monitoring; and (2) standalone app or gamification and education, defined as interventions primarily relying on automated algorithms, self-guided goal setting, gamification, or one-way educational pushes without regular clinical supervision of exercise intensity. Assessment of publication bias was restricted to visual inspection of the funnel plot for outcomes reported by 10 or more studies [42]. The certainty of evidence for each outcome was evaluated using the GRADE (Grading of Recommendations Assessment, Development and Evaluation) approach [43] (Multimedia Appendix 3). Statistical significance was set at *P*<.05. Additionally, granular device-level specifications for physical activity metrics—including tracking brands, anatomical wear locations, and minimum wear-time validation criteria—were systematically extracted and categorized in Multimedia Appendix 4 [22,26,30] to evaluate cross-study technical homogeneity.

## Results

### Study Selection

**Figure 1.**
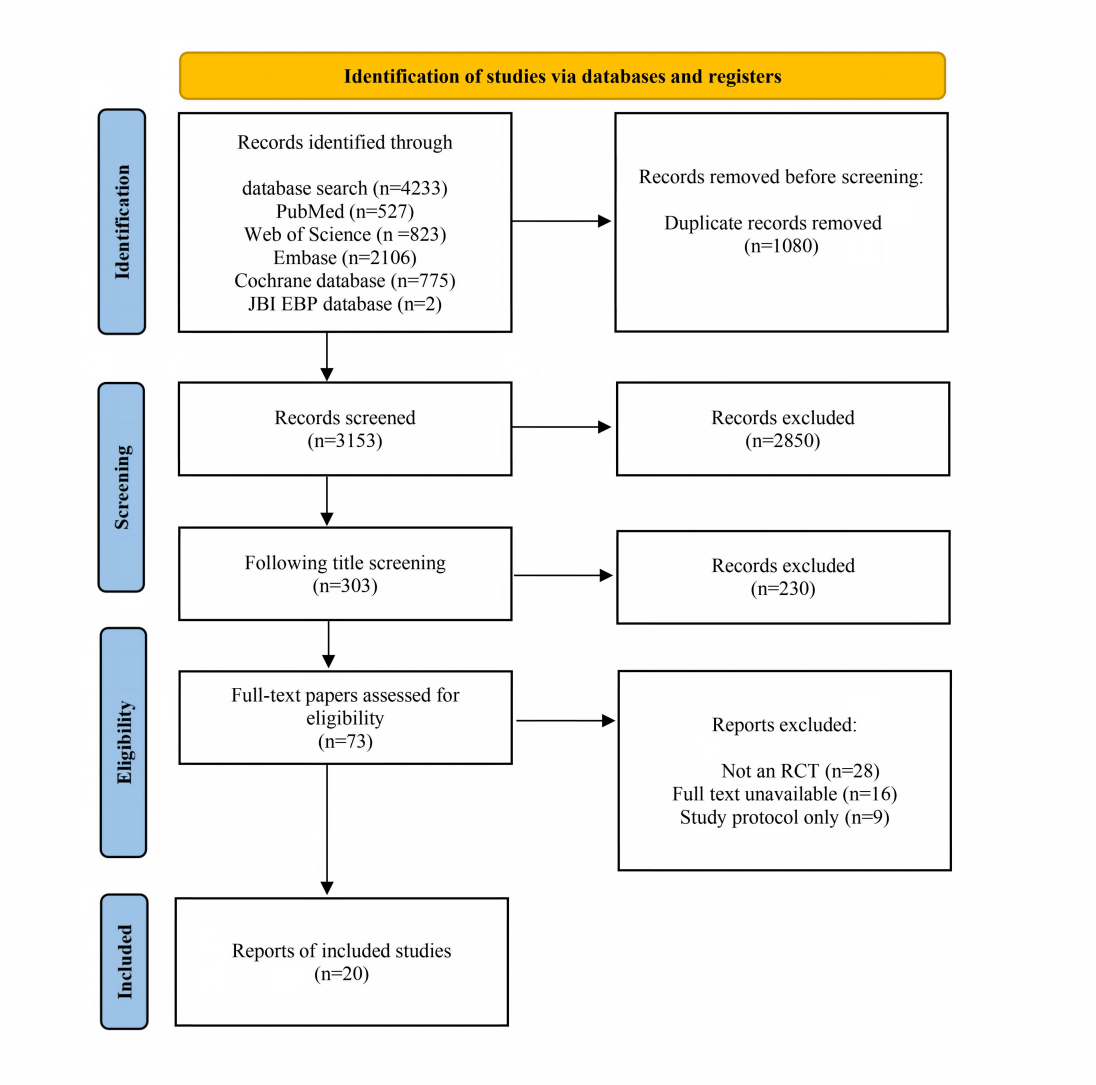
PRISMA flow diagram of study selection. JBI EBP: Joanna Briggs Institute Evidence-Based Practice; PRISMA: Preferred Reporting Items for Systematic Reviews and Meta-Analyses; RCT: randomized controlled trial.

The initial electronic search identified 4233 records. After removing 1080 duplicates, 3153 records were screened based on titles and abstracts. A total of 3080 records were excluded during this phase, leaving 73 full-text papers to be assessed for eligibility. Of these, 53 were subsequently excluded for the following reasons: the study was not an RCT (n=28), the full text was unavailable (n=16), or the publication was a study protocol without results (n=9). Ultimately, 20 studies met the inclusion criteria and were included in the systematic review. The detailed selection process is depicted in the PRISMA flow diagram (Figure 1).

### Study Characteristics

A total of 20 eligible publications were included in this systematic review. Following the PRISMA guidelines to prevent double-counting of participants, multiple reports originating from the same study population were grouped, resulting in 16 unique RCTs involving a total of 3441 participants included in the final analysis. For example, multiple publications from the TELEREH-HF (Telerehabilitation in Heart Failure Patients) trial and the IT IS HOPE 4 HF (Interval Training Versus High-Intensity Interval Training or Moderate Continuous Training in Heart Failure Patients) trial were respectively synthesized as single study units [22-41]. Across these 16 unique trials, sample sizes varied widely from pilot studies to large-scale multicenter trials (ranging from 20 to 850 participants). The trials were geographically diverse, encompassing populations from North America (the United States and Canada), Europe (Germany, Poland, Norway, Sweden, Belgium, and Italy), the Middle East (Jordan), and the Asia-Pacific region (China, Japan, and Australia). Participants were predominantly older adults (mean age typically ranging from 59 to 78 years) diagnosed with New York Heart Association class I-IV heart failure, encompassing both reduced and preserved ejection fractions.

The interventions used a broad spectrum of DHTs. While most relied on mobile apps, web-based platforms, and telemonitoring [22-35], innovative modalities such as virtual reality (exergaming) were also used. Notably, several interventions were grounded in established behavioral theories, including cognitive behavioral therapy, the Fogg behavior model, and the theory of planned behavior, to enhance adherence and outcomes. Intervention durations ranged from 8 weeks to 26 months, with follow-up periods extending up to 24 months in some trials. Detailed baseline characteristics of the study populations are presented in Table 1. Furthermore, a systematic breakdown of the specific active ingredients for each intervention—including the presence or absence of exercise prescriptions, remote supervision modalities, symptom monitoring, and the incorporation of behavioral theories or gamification—is provided in the intervention component matrix (Multimedia Appendix 2).

**Table 1.** Characteristics of included studies^a^.

Study or trial name	Participants and clinical setting	Intervention components and DHT^b^ type	Outcomes and time points
Radhakrishnan et al (2021) [33]	n=38 (intervention: n=19; control: n=19) Age: ≥55 years NYHA^c^: II-III Setting: Cardiac rehabilitation and clinics, United States	DHT type: Sensor-controlled digital game app+Bluetooth scale+tracker Components: Gamified rewards, real-time behavioral feedback, daily weight, and activity tracking	Outcomes: Physical activity steps, daily weight monitoring, KCCQ^d^, and HF^e^ knowledge Time points: Baseline, 6, 12, and 24 weeks
TARGET-HF-DM^f^ trial (Felker et al, 2022 [30])	n=187 (intervention: n=92; control: n=95) Age: mean 59 (SD 11) years NYHA: II-IV (with diabetes) Setting: 6 clinical sites, United States	DHT type: Mobile health (mHealth) app (D-3 Pillbox)+wearable tracker (Withings Go) Components: Automated personalized text messages, step tracking, and medication skill-building	Outcomes: Daily step count, KCCQ-OSS^g^, medication adherence, and NT-proBNP^h^ Time points: Baseline, 3, and 6 months
HITS^i^ trial (Lödding et al, 2024 [24])	n=699 (intervention: n=344; control: n=354) Age: 18-68 years NYHA: I-III Setting: Multiple centers, Germany	DHT type: HITS Tablet app+wearable tracker+HR^j^ or BP^k^ monitors+smart scale Components: Home-based exercise videos, telemonitoring, and self-management education	Outcomes: Physical activity (MET^l^*hours per week), daily steps, and completed exercise videos Time points: Baseline, 6, and 12 months
TELEREH-HF^m^ trial (Piotrowicz et al, 2020 [38]; Szalewska et al, 2021 [37]; Piotrowicz et al, 2022 [29])	n=850 (intervention: n=425; control: n=425) Age: 18-62 years NYHA: I-III (LVEF^n^≤40%) Setting: 5 centers, Poland	DHT type: Telerehabilitation set (EHO^o^ mini device) for tele-ECG^p^ and BP Components: 9-week hybrid comprehensive telerehabilitation, remote monitoring, and tele-psychology	Outcomes: Days alive or out of hospital, mortality, 6MWD^q^, peak VO_2_^r^, and depression (BDI-II^t^) Time points: Baseline, 9 weeks, and 14-26 months
CHF-CePPORT^s^ trial (Nolan et al, 2021 [34])	n=231 (intervention: n=117; control: n=114) Age: 18-59 years NYHA: I-III (LVEF≤45%) Setting: 3 tertiary hospitals, Canada	DHT type: Internet-based e-counseling program+activity monitor Components: Motivational or cognitive behavioral tools, self-assessment trackers, and multimedia videos	Outcomes: KCCQ-OSS^u^, 6MWT^v^, 4-day step count, program engagement, and diet Time points: Baseline, 4, and 12 months
IT IS HOPE 4 HF^w^ trial (Lundgren et al, 2025 [23]; Lundgren et al, 2023 [28]; Langlo et al, 2022 [31])	n=61 (intervention: n=31; control: n=30) Age: 18-68 years NYHA: II-III Setting: 2 outpatient clinics, Norway	DHT type: Real-time videoconferencing software via tablet Components: Home-based high-intensity interval training and live group-based remote supervision	Outcomes: MVPA^x^, peak VO_2_, 6MWT, QoL^y^ (MLHFQ^z^ and EQ-5D), and adherence Time points: Baseline, 3, 6, 12, and 24 months
Saleh et al (2022) [26]	n=132 (intervention: n=65; control: n=67) Age: mean 60.8 (SD 10.5) years NYHA: I-III Setting: Outpatient clinics, Jordan	DHT type: Samsung health app+social media Components: Sedentary behavior interruption, daily step goals, and multimedia messaging	Outcomes: Daily step counts, HF symptom burden, and HRQoL^aa^ (SF-36^ab^) Time points: Baseline, 2, and 8 weeks
Jaarsma et al (2021) [36]	n=605 (intervention: 305; control: 300) Age: 18-67 years NYHA: I-IV Setting: 10 HF centers, 6 countries	DHT type: Exergame (Nintendo Wii Sports) Components: Home-based virtual reality gaming (tennis and bowling) and telephone motivational support	Outcomes: 6MWT, muscle function, exercise self-efficacy, and physical activity Time points: Baseline, 3, 6, and 12 months
Song et al (2024) [25]	n=200 (intervention: n=100; control: n=100) Age: 18-63 years NYHA: II-IV Setting: Grade A hospital, China	DHT type: “Health & Happiness” chronic disease management app Components: “Hospital-to-home+online-to-offline” (H2H+O2O) scheme, symptom self-test, and point-prize incentive	Outcomes: BNP^ac^, LVEF, 6MWT, HF knowledge, self-care behavior (SCHFI^ad^) Time points: Baseline and 3 months after discharge
Nagatomi et al (2022) [32]	n=30 (intervention: n=15; control: n=15) Age: mean 63.7 (SD 10.1) years (frail) NYHA: II-III Setting: Kyushu University, Japan	DHT type: Wearable tracker (Fitbit Inspire HR)+smartphone app Components: ICT^ae^-based exercise and nutrition guidance, weekly message feedback, and meal photo analysis	Outcomes: 6MWD, muscle strength, BNP, KCCQ, and physical frailty Time points: Baseline and 3 months
Peng et al (2018) [39]	n=98 (intervention: n=49; control: n=49) Age: 18-66 years NYHA: I-III Setting: Teaching hospital, China	DHT type: Instant messaging (QQ and WeChat) via smartphone Components: Online webcam supervision, resistance or endurance training, and remote consultations	Outcomes: QoL (MLHFQ), 6MWD, resting HR, HADS^af^, and LVEF Time points: Baseline, 2, and 6 months
Hwang et al (2017) [40]	n=53 (intervention: n=24; control: n=29) Age: 18-67 years NYHA: II-III Setting: 2 tertiary hospitals, Australia	DHT type: Online videoconferencing software via laptop computer Components: 12-Week real-time group-based exercise and education delivered to homes	Outcomes: 6MWD, TUGT^ag^ balance, quadriceps strength, and QoL (MLWHFQ and EQ-5D) Time points: Baseline, 12, and 24 weeks
Smolis-Bąk et al (2015) [41]	n=52 (intervention: n=26; control: n=26) Age: 18-62 years NYHA: III (with CRT-D^ah^) Setting: Institute of Cardiology, Poland	DHT type: Telemonitoring ECG via cell phone Components: 8-Week home-based exercise training with active telemonitoring guidance	Outcomes: Peak VO_2_, AT^ai^, 6MWT, LVEF, QoL (NHP^aj^), and depression (BDI^ak^) Time points: Baseline, 3-4, and 12 months
HeartMan trial (Clays et al, 2021 [35])	n=56 (intervention: n=34; control: n=22) Age: 18-63 years NYHA: II-III (LVEF≤40%) Setting: 4 hospitals, Belgium or Italy	DHT type: Mobile app+sensing wristband+BP or weight scale Components: Decision support system, physical exercise, cognitive behavioral therapy, psychological support, and nutrition	Outcomes: HRQoL (MLHFQ), self-care (SCHFI), 6MWT, and illness perception (IPQ^al^) Time points: Baseline and 3-6 months
HEALTHY Pilot (Blomqvist et al, 2025 [22])	n=20 (intervention: n=10; control: n=10) Age: 18-78 years NYHA: II-III Setting: 5 primary care centers, Sweden	DHT type: Activity Coach app module on Optilogg mHealth tool+ActiGraph Components: Daily physical activity tracking, personalized goal setting, and educational tips	Outcomes: Subjective goal attainment, HRQoL (KCCQ), and accelerometer steps or sedentary time Time points: Baseline and 12 weeks
Wang and Zhu (2023) [27]	n=129 (intervention: n=65; control: n=64) Age: 18-61 years NYHA: II-IV Setting: Cardiovascular department, China	DHT type: WeChat-based public number and dedicated group chat Components: “Hospital-home” integrated health education and self-monitoring picture feedback	Outcomes: Self-efficacy (GSES^am^), self-management, MLHFQ and 6MWT Time points: Baseline and 3 months

a The 20 included publications represent 16 unique randomized controlled trials. Multiple reports originating from the same trial (eg, TELEREH-HF and IT IS HOPE 4 HF) were grouped as a single study unit to prevent double-counting of participants.

b DHT: digital health technology.

c NYHA: New York Heart Association.

d KCCQ: Kansas City Cardiomyopathy Questionnaire.

e HF: heart failure.

f TARGET-HF-DM: Targeted Automated Text Messaging to Improve Physical Activity and Heart Failure Outcomes in Patients With Heart Failure and Diabetes Mellitus.

g KCCQ-OSS: Kansas City Cardiomyopathy Questionnaire Overall Summary Score.

h NT-proBNP: N-terminal pro-B-type natriuretic peptide

i HITS: Heart Failure Information Technology Support.

j HR: heart rate.

k BP: blood pressure.

l MET: metabolic equivalent of task.

m TELEREH-HF: Telerehabilitation in Heart Failure Patients.

n LVEF: left ventricular ejection fraction.

o EHO: Electro-Hospital-Oyster (telemonitoring system device).

p ECG: electrocardiogram.

q 6MWD: 6-minute walk distance.

r Peak VO_2_: peak oxygen uptake.

s CHF-CePPORT: Chronic Heart Failure Canadian e-Health Patient-Centered Portal to Optimize Health Outcomes.

t BDI-II: Beck Depression Inventory-II.

u KCCQ-OS: Kansas City Cardiomyopathy Questionnaire Overall Summary score.

v 6MWT: 6-minute walk test.

w IT IS HOPE 4 HF: Interval Training Versus High-Intensity Interval Training or Moderate Continuous Training in Heart Failure Patients.

x MVPA: moderate-to-vigorous physical activity.

y QoL: quality of life.

z MLHFQ: Minnesota Living with Heart Failure Questionnaire.

aa HRQoL: health-related quality of life.

ab SF-36: 36-Item Short Form Health Survey.

ac BNP: B-type natriuretic peptide.

ad SCHFI: Self-Care of Heart Failure Index.

ae ICT: information and communication technology.

af HADS: Hospital Anxiety and Depression Scale.

ag TUGT: Timed Up and Go Test.

ah CRT-D: cardiac resynchronization therapy defibrillator.

ai AT: anaerobic threshold.

aj NHP: Nottingham Health Profile.

ak BDI: Beck Depression Inventory.

al IPQ: Illness Perception Questionnaire.

am GSES: General Self-Efficacy Scale.

### Risk of Bias in Included Studies

**Figure 2.**
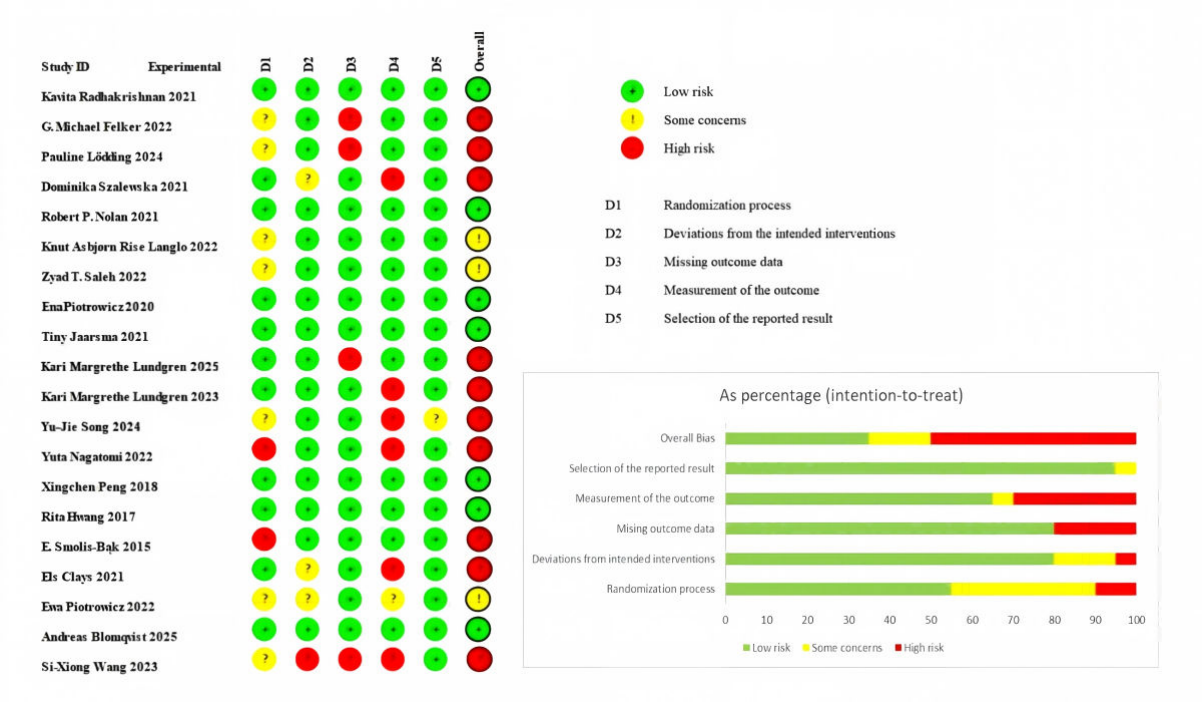
Risk of bias summary [22-41].

We assessed the risk of bias for the 20 included RCTs using the Cochrane Risk of Bias tool version 2 [44]. As depicted in Figure 2, a total of 7 (35%) trials demonstrated a low overall risk of bias, whereas 10 (50%) were classified as high risk, and 3 (15%) raised some concerns. The high risk of bias was predominantly driven by issues in outcome measurement (domain 4) and missing outcome data (domain 3). Specifically, the inherent nature of digital health interventions often precluded participant blinding, making subjective outcomes such as quality of life susceptible to detection bias. Furthermore, several studies were flagged for high attrition rates or insufficient handling of missing data, including the absence of intention-to-treat analysis. Uncertainties regarding the randomization process (domain 1) also affected specific trials. Despite these limitations, the dataset was considered sufficient for robust synthesis. To definitively ensure that these methodological flaws did not artificially drive our primary findings, we performed a prespecified sensitivity analysis for the primary functional outcome (6MWD), as detailed in the Risk of Bias in Included Studies section. Nevertheless, to maintain maximum methodological rigor, the overarching impact of the open-label designs was formally integrated into our evidence grading by downgrading the certainty of evidence.

### Effects of DHT Interventions

#### 6MWD in Standardized Tests

**Figure 3.**
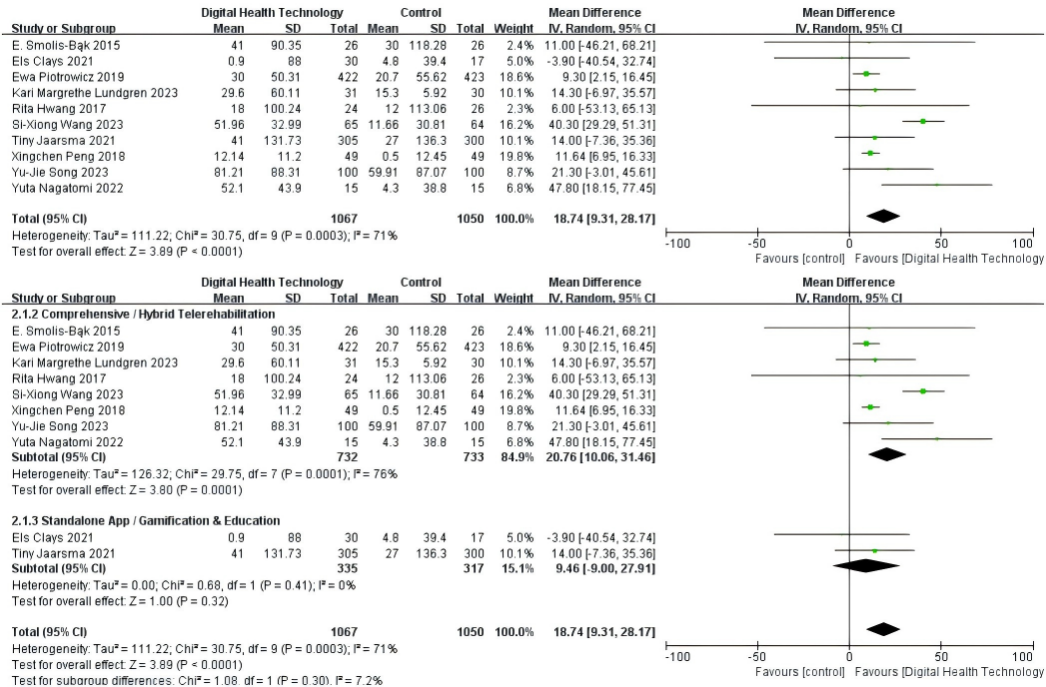
Analysis of 6-minute walk distance outcome [25,27,28,32,35,36,38-41].

In total, 10 studies involving 2117 participants assessed the effects of DHTs on the 6MWD. The pooled analysis using a random-effects model demonstrated that DHT interventions significantly increased 6MWD compared with usual care (MD 18.74 m, 95% CI 9.31‐28.17; *P*<.001), with substantial statistical heterogeneity observed (*I*^2^=71%; *P*<.001; Figure 3). To investigate the sources of this heterogeneity, we performed a subgroup analysis based on the intervention mode. Applying the predefined operational criteria, the subgroup of comprehensive or hybrid telerehabilitation (8 cohorts) demonstrated a significant and clinically relevant benefit favoring the intervention (MD 20.76 m, 95% CI 10.06 to 31.46; *P*=.001). Although statistical heterogeneity was observed in this subgroup (*I*^2^=76%), visual inspection of the forest plot reveals that this inconsistency pertains strictly to the magnitude of the benefit rather than the direction. All hybrid interventions consistently favored the treatment arm; however, recent Asian trials using high-frequency smartphone integrations [27,32] reported substantially larger effect sizes (MD >40 m) compared to earlier European trials using traditional tele-electrocardiogram monitoring [38]. Conversely, the subgroup using standalone apps and automated education (2 cohorts) showed a markedly diluted and nonsignificant effect (MD 9.46 m, 95% CI –9.00 to 27.91; *P*=.32) with 0% heterogeneity. These findings, derived after meticulously eliminating double-counting, structurally validate that closed-loop human supervision in hybrid models primarily drives the physiological benefits. The certainty of the evidence for this outcome was graded as moderate according to the GRADE assessment (Multimedia Appendix 3). Visual inspection of the funnel plot did not reveal substantial asymmetry, suggesting a low risk of publication bias (Multimedia Appendix 5). Furthermore, to ensure that methodological flaws did not artificially drive these findings, we performed a prespecified sensitivity analysis excluding the trials classified as having a high risk of bias or some concerns. The removal of these trials yielded a pooled effect size that remained highly significant in favoring DHT interventions (MD 11.01 m, 95% CI 7.17 to 14.86; *P*<.001). Remarkably, this exclusion completely resolved the statistical heterogeneity (*I*^2^=0%; *P*=.94), explicitly indicating that the lower-quality studies were the primary drivers of the previously observed variance (Multimedia Appendix 6) [25,27,28,32,35,36,38-41]. This confirms that the functional benefits are highly stable and fundamentally reliable.

#### Peak VO_2_ Assessed by Cardiopulmonary Exercise Testing

**Figure 4.**
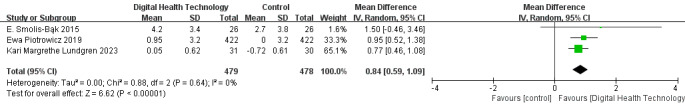
Analysis of peak oxygen uptake outcome [28,38,41].

To rigorously evaluate maximal aerobic capacity while preventing unit-of-analysis errors, we restricted our meta-analysis to the primary overall cohorts from 3 independent trials, yielding a clean and robust sample of 957 participants. The pooled analysis using a random-effects model revealed a highly consistent and statistically significant improvement, with DHT interventions increasing peak VO_2_ by 0.84 mL/kg/minute compared with controls (95% CI 0.59‐1.09; *P*<.001). Notably, statistical heterogeneity was entirely absent across these trials (*I*^2^=0%; *P*=.64; Figure 4), and visual inspection of the forest plot confirmed that all individual cohorts confidently favored the intervention group. These methodologically corrected findings firmly establish that DHT interventions reliably improve maximal cardiorespiratory fitness in adults with CHF. The certainty of this evidence was graded as moderate according to the GRADE assessment.

#### Objectively Measured Daily Step Count

In total, 3 studies involving 301 participants assessed the effects of DHT interventions on daily step counts. Due to the extreme statistical heterogeneity (*I*^2^=99%; *P*<.001) and evident clinical diversity among the trials, a pooled meta-analysis was deemed inappropriate, and a narrative synthesis was conducted instead, with the unpooled forest plot provided for transparency (Multimedia Appendix 7) [22,26,30]. This profound divergence is methodologically explained by substantial variations in hardware infrastructure and anatomical wear locations across the trials, ranging from wrist-worn commercial trackers [30] and pocket-carried smartphone apps [26] to research-grade hip-worn accelerometers [22]. Consequently, the outcomes varied widely at the study level, reflecting not only differences in baseline physical activity or intervention intensity but also instrument-driven variances in motion-sensing thresholds. For instance, Saleh et al [26] reported a substantial and statistically significant increase in daily steps, which may be attributed to their intensive, gamified goal-setting approach combined with a highly sedentary baseline population. Conversely, trials such as Felker et al [30] and Blomqvist et al [22] observed minimal or nonsignificant changes, potentially reflecting a ceiling effect where participants were either already moderately active or where the intervention relied on passive tracking rather than proactive behavioral nudges. Consequently, current evidence does not demonstrate a consistent or uniform benefit of DHT interventions on objectively measured daily step counts. The overall certainty of evidence for this outcome was graded as very low due to serious risk of bias, very serious inconsistency across study results, and imprecision.

#### Health-Related Quality of Life

**Figure 5.**
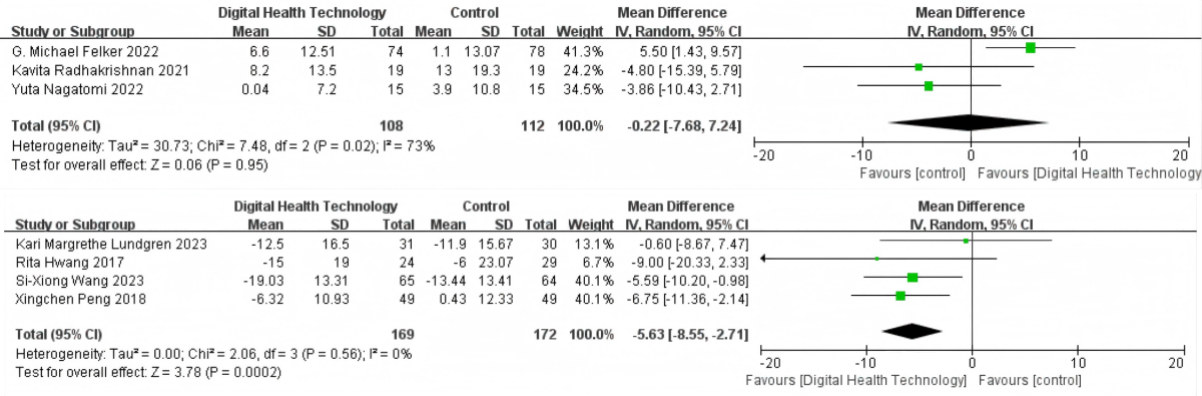
Analysis of health-related quality of life outcome [27,28,30,32,33,39,40].

We evaluated the effects of DHT interventions on disease-specific health-related quality of life (HRQoL) using the MLHFQ and the KCCQ. For the MLHFQ, where lower scores indicate better health status, the pooled analysis of 4 studies involving 341 participants demonstrated that DHT interventions yielded a statistically significant improvement in quality of life compared with controls, with a mean reduction of 5.63 points (95% CI –8.55 to –2.71; *P*<.001; Figure 5). Notably, statistical heterogeneity was absent (*I*^2^=0%; *P*=.56), indicating a highly consistent beneficial effect across these trials, and the certainty of this evidence was graded as moderate. In contrast, the analysis of 3 studies involving 220 participants using the KCCQ (where higher scores indicate better status) revealed no significant difference between the DHT and control groups (MD –0.22 points, 95% CI –7.68 to 7.24; *P*=.95). Substantial heterogeneity was observed for this outcome (*I*^2^=73%; *P*=.02), reflecting inconsistent treatment effects across the trials, and the certainty of the evidence was graded as very low due to inconsistency and imprecision. To investigate the source of this substantial heterogeneity, we performed a sensitivity analysis stratifying the KCCQ trials by comparator type (true usual care vs enhanced usual care). The pooled effect within the “enhanced usual care” subgroup remained nonsignificant (MD 1.56, 95% CI –8.25 to 11.37; *P*=.76) with high residual heterogeneity (*I*^2^=68%). Within this subgroup, Radhakrishnan et al [33] demonstrated a profound dilution effect, as their active control arm achieved a 13-point increase in KCCQ score, effectively neutralizing the intervention’s apparent benefit. Meanwhile, the single trial using “true usual care” [32] also yielded a nonsignificant effect (MD –3.86, 95% CI –10.43 to 2.71; *P*=.25; Multimedia Appendix 8) [30,32,33]. These findings suggest that the heterogeneity in general quality-of-life outcomes is driven by highly variable control group designs as well as specific patient demographic factors. Overall, while MLHFQ data consistently supported the positive impact of DHTs, KCCQ results remained inconclusive.

#### Safety and AE Reporting

Several included trials explicitly reported on AEs associated with home-based DHT interventions, though the collection methodologies varied significantly. Systematic AE collection was rigorously used in several trials. For instance, Lundgren et al [23] defined exercise-related AEs as any unwanted medical event during sessions, systematically recording them via supervising therapists, structured physician interviews at follow-up, and thorough audits of hospital records to capture unreported events. Similarly, Hwang et al [40] used a predefined checklist of potential AEs attached to exercise recording forms, which were systematically tallied by blinded assessors after each supervised session. Piotrowicz et al [38] (TELEREH-HF) actively monitored for AEs during and up to 1 hour directly following telemonitored exercise, while Nagatomi et al [32] systematically recorded all AEs and mandated that attending cardiologists formally adjudicate their causal relationship to the intervention.

Conversely, passive or hybrid collection methods were used in other studies. Jaarsma et al [36] (HF-Wii) documented AEs either as spontaneously reported by patients or incidentally observed by the research team during the trial. Peng et al [39] relied predominantly on passive safety monitoring, instructing patients to independently cease exercise and report to a physician only if they experienced signs of physical distress (eg, chest pain and severe weakness). Reassuringly, across both systematic and passive reporting methodologies, no severe intervention-related AEs (such as death, syncope, or cardiac arrest during active device use) were reported, confirming the overall safety profile of home-based digital health interventions in patients with heart failure.

## Discussion

### Principal Findings

This systematic review and meta-analysis synthesized evidence from 16 unique RCTs (across 20 publications) to evaluate the efficacy of DHT interventions in adults with CHF. Our findings demonstrate that DHT interventions yield robust and consistent improvements in physiological outcomes, specifically functional exercise capacity (6MWD) and cardiorespiratory fitness (peak VO_2_), with moderate-certainty evidence. In contrast, the effects on daily physical behavior (step counts) and general health status (KCCQ) appeared less consistent. While physiological capacity improved reliably, evidence for sustained behavior change (ie, increased daily steps) is currently limited to a very small number of trials with extreme methodological diversity. Therefore, any statements regarding the translation of physiological gains into increased daily activity must be made with caution. The current limited evidence base suggests that improved fitness does not automatically guarantee changes in daily habits, highlighting a significant gap in the literature. This aligns with the well-documented “intention-behavior gap” in physical activity research, demonstrating that physiological readiness often fails to translate into autonomous daily exercise without continuous, context-specific behavioral nudges [45].

A key insight from our subgroup analysis is that successful DHT interventions are not merely about passive tracking but rather about establishing a closed-loop clinical management system. The superior efficacy of comprehensive hybrid models can be attributed to several implementation mechanisms facilitated by technology. First, the integration of real-time monitoring creates a “digital safety net” that reduces kinesiophobia, a common barrier in patients with heart failure, thereby encouraging adherence to higher-intensity exercise prescriptions [46]. Furthermore, DHTs bridge the vulnerable phase postdischarge by transforming episodic hospital care into continuous home-based management [47,48]. As evidenced by “hospital-to-home” and “online-to-offline” models, continuous digital interaction via platforms such as WeChat allows specialist nurses to stratify patients before discharge and provide seamless remote guidance. This continuity enhances self-efficacy and symptom recognition, contrasting sharply with the fragmentation often seen in standard care. Finally, interventions incorporating active engagement strategies leverage behavioral economics principles to sustain motivation, addressing the engagement drop-off often observed in standalone app-based approaches [49].

A critical distinction emerging from our analysis is the divergence between improving functional capacity and daily behavioral performance. Regarding functional capacity, our results demonstrated uniform robustness. Based on our methodologically corrected cohorts, the results confirmed that peak VO_2_ improved significantly and consistently across all independent cohorts (MD 0.84 mL/kg/minute; *I*^2^=0%). Concurrently, 6MWD showed significant gains, primarily driven by structured hybrid telerehabilitation models [23,38,41]. These interventions are based on a rigorous clinical logic involving inpatient initiation followed by home-based execution. Crucially, many of these hybrid models used specialized tele-electrocardiogram monitoring devices that allow clinicians to view patient data in real time or immediately after exercise. This technology acts as a safety net, giving clinicians the confidence to prescribe and supervise higher-intensity training in a home setting, thereby directly enhancing physiological adaptations. By contrast, as detailed in our intervention component matrix (Multimedia Appendix 2), interventions relying solely on passive behavioral nudges, gamification, or automated decision support systems without human-in-the-loop oversight [35] failed to elicit significant physiological adaptations. This systematic component breakdown confirms that while digital tools successfully facilitate health care access, the critical “active ingredients” for substantial physical capacity improvement remain a structured exercise prescription coupled with professionally supervised exercise intensity and robust remote monitoring. Crucially, the magnitude of these functional gains must be contextualized against minimal clinically important difference (MCID) thresholds rather than statistical significance alone. The mean improvement of 20.76 m in the 6MWD achieved by hybrid models falls squarely within the recognized MCID range of 14-30.5 m for adults with cardiovascular pathology [50], translating to noticeable improvements in patients’ ability to perform routine daily tasks. Furthermore, while the overall 0.84 mL/kg/minute increase in peak VO_2_ may seem numerically modest, it approaches the established threshold (eg, a modest increase of ~6% or 1.0 mL/kg/minute) that has been extensively correlated with better clinical outcomes and reduced mortality in major trials such as HF-ACTION (Heart Failure: A Controlled Trial Investigating Outcomes of Exercise Training) [51], underscoring the profound clinical utility of these structured digital interventions.

Regarding daily behavioral performance, the evidence base is currently too small and methodologically inconsistent to draw definitive conclusions. While some high-intensity gamified interventions [26,33] achieved clinically meaningful increases in daily steps, other studies [22] found no effect. Rather than a uniform treatment effect, this discrepancy highlights the complexity of behavior change. We hypothesize that dynamic adjustment and social integration in app design might be critical factors, though more robust behavioral trials are required to confirm this. For example, in the study by Nagatomi et al [32], physical therapists used Fitbit data to adjust training intensity weekly based on patient condition, whereas standard apps often rely on static goals that fail to adapt to fluctuating symptoms. Moreover, successful interventions often incorporated gamification and social interaction (eg, Jaarsma et al’s [36] exergaming and Wang and Zhu’s [27] group chats) to address psychological barriers such as boredom and isolation. Crucially, the data-capture mechanism itself introduces a distinct confounding layer: research-grade hip accelerometers (eg, ActiGraph in Blomqvist et al [22]) provide passive, unbiased tracking of all baseline movement but lack interactive behavior-change cues, whereas interactive smartphone apps [26] successfully drive targeted behavioral compliance through gamification but inherently miss ambient steps if the device is not continuously carried in the pant pocket. Without these active ingredients, mere tracking may lead to a ceiling effect, particularly in older or frail populations facing physical limitations or digital barriers [52]. Crucially, despite varying efficacy on behavior, the included trials reported no significant increase in AEs associated with home-based DHT interventions. This reinforces that remote rehabilitation is a safe alternative to center-based care, provided it is supported by technologies allowing for symptom-based feedback loops or real-time supervision [23].

The complexity of interpreting DHT efficacy is further illustrated by the divergence in HRQoL outcomes. We observed highly consistent benefits when assessing symptom burden via the MLHFQ (*I*^2^=0%). Notably, the pooled reduction of 5.63 points directly exceeds the 5-point MCID threshold endorsed for this specific instrument in contemporary heart failure outcome reviews [53], demonstrating that communication-based DHTs not only yield statistically significant results but also reliably deliver clinically meaningful reductions in daily symptom burden, regardless of the control setting. By contrast, results for broader quality of life measured by the KCCQ were conflicting. Based on our sensitivity evaluation, this inconsistency is fundamentally driven by a combination of comparator heterogeneity and patient vulnerability. The “dilution effect” is particularly evident in studies like Radhakrishnan et al [33], where control participants received “technology-enhanced usual care” (eg, smart weighing scales and activity trackers). These active elements artificially elevated the health awareness and baseline performance of the control arms, effectively neutralizing the comparative intervention effect. However, comparator type alone does not explain all variance; the null finding in Nagatomi et al [32], despite their use of true usual care, highlights that populations with physical frailty may face physiological and psychological barriers that prevent rapid improvements in general quality of life metrics like the KCCQ. This underscores that when DHTs are evaluated, future trial designs must carefully balance the ethical need for active controls against the statistical risk of effect dilution. Additionally, technical burdens such as syncing failures or connectivity issues in intervention arms can paradoxically reduce quality of life and negate potential benefits. Therefore, future clinical applications should prioritize user-centered designs that minimize technical friction and integrate behavioral interventions, such as cognitive behavioral therapy or motivational interviewing algorithms, directly into the app interface to address the holistic psychosocial dimensions of heart failure.

Compared to previous meta-analyses that broadly assessed telemedicine for heart failure by focusing on mortality and hospitalization, our study offers unique value by dissecting the specific impact on physical outcomes and the implementation modes behind them. While earlier reviews often grouped simple telephone support with complex telerehabilitation, our stratification into comprehensive hybrid models versus app-based education models clarifies that structure and supervision intensity are critical factors for physiological improvement. Furthermore, by restricting our scope to the last decade, our review captures the technological shift from traditional landline-based systems to modern smartphone apps and wearables, providing evidence directly applicable to contemporary clinical practice.

### Clinical Implications and Future Directions

For clinical practice, our findings advocate a stratified approach based on specific therapeutic goals. To improve functional capacity, clinicians should prioritize hybrid models combining remote monitoring with structured exercise intensity. In this context, DHTs function as “digital safety nets” rather than simple trackers, enabling the safe prescription of higher-intensity training in home settings. Conversely, for symptom management and reducing disease burden, scalable mobile apps or messaging platforms using integrated care models offer effective, low-intensity alternatives. However, caution is warranted for frail or older populations, where standard digital interventions often encounter adherence barriers. For these patients, tailored designs prioritizing usability and lower-intensity maintenance are essential to avoid the ceiling effect of passive monitoring.

Future research must move beyond simple efficacy trials to investigate optimal delivery methods for specific subgroups. Specifically, next-generation DHTs should leverage AI to analyze real-time wearable data and dynamically adjust exercise prescriptions. This would transform static exercise goals into adaptive regimens responding to the patient’s daily physiological state. Furthermore, to sustain long-term engagement and combat the high attrition rates common in digital health, future apps should integrate sophisticated gamification and social interaction features. Hybrid models involving periodic human check-ins may remain necessary to maintain long-term adherence, as fully automated systems frequently experience user disengagement over time. Finally, extended follow-up periods are needed to assess whether digital engagement and physical benefits persist or wane over years.

Finally, real-world implementation of closed-loop DHTs must navigate complex ethical and equity landscapes. As synthesized by Veras et al [54], deployment risks exacerbating disparities if access to connectivity and compatible devices remains unequal. Digital literacy barriers are particularly pronounced among older adults and those with physical impairments. Therefore, systems must prioritize accessible, user-centered design and provide robust onboarding. Furthermore, the generation of vast sensitive physiological data necessitates stringent cybersecurity and transparent consent governance. Crucially, clinical workflows must clearly delineate safety monitoring responsibilities—defining who responds to real-time alerts and when—to ensure patient safety without creating liability ambiguities or overwhelming clinician capacity [54].

### Limitations

This study has several limitations. First, despite subgroup analyses, heterogeneity remained high for step counts and KCCQ outcomes. This reflects the inherent diversity in DHT modalities, ranging from simple SMS text messaging to complex sensor-based systems. Second, the majority of included studies were open-label due to the nature of the interventions, introducing a risk of performance and detection bias for subjective outcomes like HRQoL. Third, female patients were underrepresented in the included trials, comprising approximately 24% of the population. This limits the generalizability of our findings to female patients who may face distinct barriers to digital adoption. Finally, variation in standard care across different health care systems likely influenced relative effect sizes, as some control groups received enhanced monitoring (technology-enhanced usual care) that might underestimate the true benefit of digital interventions. Finally, publication bias could only be reliably assessed for the 6MWD outcome, which met the required threshold of 10 studies and demonstrated no substantial asymmetry. For all other outcomes, including peakVO_2_, daily steps, MLHFQ, and KCCQ, the inclusion of fewer than 10 studies precluded a robust evaluation via funnel plots, meaning that potential publication bias for these specific metrics cannot be entirely ruled out.

### Conclusions

DHTs, particularly in the form of comprehensive hybrid telerehabilitation, are safe and effective strategies to improve cardiorespiratory fitness, functional exercise capacity, and symptom-related quality of life in patients with CHF. However, evidence regarding their impact on daily physical activity behavior and general health status remains inconsistent. This variability appears to be driven by differences in intervention intensity and the type of control group used. Therefore, to maximize clinical benefit, future clinical practice should prioritize structured, medically supervised hybrid models over standalone monitoring tools. Further research is needed to determine how to effectively translate physiological gains into sustained improvements in daily behavioral performance.

## Supplementary material

10.2196/91820 Multimedia Appendix 1 Detailed search strategies for all electronic databases.

10.2196/91820 Multimedia Appendix 2 Intervention component matrix and taxonomy.

10.2196/91820 Multimedia Appendix 3 Grading of Recommendations Assessment, Development and Evaluation assessment for certainty of evidence.

10.2196/91820 Multimedia Appendix 4 Hardware specifications and wear-time validation criteria for daily step counts.

10.2196/91820 Multimedia Appendix 5 Funnel plot for the assessment of publication bias (6MWD).

10.2196/91820 Multimedia Appendix 6 Sensitivity analysis for 6-minute walk distance (exclusion of trials with high risk of bias or some concerns).

10.2196/91820 Multimedia Appendix 7 Unpooled forest plot for objectively measured daily step counts.

10.2196/91820 Multimedia Appendix 8 Subgroup analysis of Kansas City Cardiomyopathy Questionnaire outcomes by comparator type (true usual care vs enhanced usual care).

10.2196/91820 Checklist 1 PRISMA 2020 checklist.

## Abbreviations

6MWD: 6-minute walk distance
AE: adverse event
CHF: chronic heart failure
DHT: digital health technology
GRADE: Grading of Recommendations Assessment, Development and Evaluation
HF-ACTION: Heart Failure: A Controlled Trial Investigating Outcomes of Exercise Training
HRQoL: health-related quality of life
IT IS HOPE 4 HF: Interval Training Versus High-Intensity Interval Training or Moderate Continuous Training in Heart Failure Patients
JBI EBP: Joanna Briggs Institute Evidence-Based Practice
KCCQ: Kansas City Cardiomyopathy Questionnaire
MCID: minimal clinically important difference
MD: mean difference
MLHFQ: Minnesota Living with Heart Failure Questionnaire
peak VO_2_: peak oxygen uptake
PRISMA: Preferred Reporting Items for Systematic Reviews and Meta-Analyses
RCT: randomized controlled trial
TELEREH-HF: Telerehabilitation in Heart Failure Patients
