# Supplementary material for: Digital Health Technology for Improving Physical Function in Adults With Chronic Heart Failure: Systematic Review and Meta-Analysis of Randomized Controlled Trials
Source: J Med Internet Res. 2026 Aug 13;28:e91820. doi: 10.2196/91820 (PMC13472526; doi:10.2196/91820)
Supplement: Supplementary file 2 [file jmir-v28-e91820-s001.docx]

**Table S1. Detailed search strategies for all electronic databases.**

| **Database** | **Search Strategy** | **Articles Retrieved** |
| --- | --- | --- |
| PubMed | **#1**:"heart failure"[MeSH Terms] OR "heart failure"[Title/Abstract] OR "cardiac failure"[Title/Abstract] OR "congestive heart failure"[Title/Abstract] OR "chronic heart failure"[Title/Abstract] OR "myocardial failure"[Title/Abstract] OR "heart decompensation"[Title/Abstract] OR "HFrEF"[Title/Abstract] OR "HFpEF"[Title/Abstract]  **#2**:"Game"[Title/Abstract] OR "gaming"[Title/Abstract] OR "video"[Title/Abstract] OR "multimedia"[MeSH Terms] OR ("social media"[MeSH Terms] OR "social medium"[Title/Abstract] OR "twitter messaging"[Title/Abstract] OR "web 2.0*"[Title/Abstract] OR "WeChat"[Title/Abstract]) OR ("text messaging"[MeSH Terms] OR "messag* text"[Title/Abstract] OR "texting*"[Title/Abstract] OR "short message service"[Title/Abstract] OR "text message*"[Title/Abstract]) OR ("telemedicine"[MeSH Terms] OR "virtual medicine"[Title/Abstract] OR "mobile health"[Title/Abstract] OR "mHealth"[Title/Abstract] OR "Telehealth"[Title/Abstract] OR "eHealth"[Title/Abstract]) OR ("virtual reality"[MeSH Terms] OR "reality virtual"[Title/Abstract]) OR ("wearable electronic devices"[MeSH Terms] OR (("device s"[All Fields] OR "equipment and supplies"[MeSH Terms] OR ("equipment"[All Fields] AND "supplies"[All Fields]) OR "equipment and supplies"[All Fields] OR "Device"[All Fields] OR "instrumentation"[MeSH Subheading] OR "instrumentation"[All Fields] OR "devices"[All Fields]) AND "wearable electronic"[Title/Abstract]) OR (("electronical"[All Fields] OR "electronically"[All Fields] OR "Electronics"[MeSH Terms] OR "Electronics"[All Fields] OR "Electronic"[All Fields]) AND "device wearable"[Title/Abstract]) OR "wearable electronic device"[Title/Abstract] OR "wearable technolog*"[Title/Abstract] OR "technology wearable"[Title/Abstract] OR "wearable device*"[Title/Abstract] OR "device wearable"[Title/Abstract] OR "electronic skin"[Title/Abstract]) OR ("internet based intervention"[MeSH Terms] OR "internet based intervention*"[Title/Abstract] OR "intervention* internet based"[Title/Abstract] OR "web based intervention*"[Title/Abstract] OR "intervention* web based"[Title/Abstract] OR "online intervention*"[Title/Abstract] OR "intervention* online"[Title/Abstract] OR "internet intervention*"[Title/Abstract] OR "intervention* internet"[Title/Abstract]) OR ("mobile applications"[MeSH Terms] OR "application* mobile"[Title/Abstract] OR "mobile app*"[Title/Abstract] OR "app mobile"[Title/Abstract] OR "portable software app*"[Title/Abstract] OR "app portable software"[Title/Abstract] OR (("Software"[MeSH Terms] OR "Software"[All Fields] OR "software s"[All Fields] OR "softwares"[All Fields]) AND "app portable"[Title/Abstract]) OR "smartphone app*"[Title/Abstract] OR "app smartphone"[Title/Abstract] OR "portable electronic app*"[Title/Abstract] OR "app portable electronic"[Title/Abstract] OR "electronic app* portable"[Title/Abstract]) OR ("digital technology"[MeSH Terms] OR "digital technolog*"[Title/Abstract] OR "technolog* digital"[Title/Abstract] OR "digital electronics"[Title/Abstract] OR "electronics digital"[Title/Abstract]) OR ("artificial intelligence"[MeSH Terms] OR "artificial intelligence"[Title/Abstract] OR "AI"[Title/Abstract] OR "computational intelligence"[Title/Abstract] OR "machine intelligence"[Title/Abstract] OR "computer reasoning"[Title/Abstract] OR "computer vision system*"[Title/Abstract] OR "Computer"[Title/Abstract]) OR "knowledge representation*"[Title/Abstract]  **#3**:"walk test"[MeSH Terms] OR "6 minute walk test"[Title/Abstract] OR "six minute walk test"[Title/Abstract] OR "6 minute walk test"[Title/Abstract] OR "6-MWT"[Title/Abstract] OR "6 min walk test"[Title/Abstract] OR "KCCQ"[Title/Abstract] OR "KCCQ-12"[Title/Abstract] OR "kansas city cardiomyopathy questionnaire"[Title/Abstract] OR "accelerometer"[Title/Abstract] OR "peak oxygen consumption"[Title/Abstract] OR "cardiopulmonary exercise test"[Title/Abstract] OR "peak vo2"[Title/Abstract] OR "CPET"[Title/Abstract] OR "exercise test"[MeSH Terms] OR "quality of life"[MeSH Terms] OR "minnesota living heart failure questionnaire"[Title/Abstract] OR "MLHFQ"[Title/Abstract] OR "chronic heart failure questionnaire"[Title/Abstract] OR "CHFQ"[Title/Abstract] OR "exercise ability"[Title/Abstract] OR "exercise capacity"[Title/Abstract] OR "quality of life"[Title/Abstract] OR "health related quality of life"[Title/Abstract] OR "QoL"[Title/Abstract]  **#4**:#1 AND #2 AND #3 | 527 |
| EMBASE | **#1** 'artificial intelligence'/exp  **#2** 'intelligence, artificial':ab,ti,kw OR 'computational intelligence':ab,ti,kw OR 'intelligence, computational':ab,ti,kw OR 'machine intelligence':ab,ti,kw OR 'computer reasoning':ab,ti,kw OR (ai:ab,ti,kw AND 'artificial intelligence':ab,ti,kw) OR 'computer vision system*':ab,ti,kw OR 'system*, computer vision':ab,ti,kw OR 'vision system*, computer':ab,ti,kw OR ('knowledge acquisition':ab,ti,kw AND computer:ab,ti,kw) OR ('acquisition, knowledge':ab,ti,kw AND computer:ab,ti,kw) OR 'knowledge representation*':ab,ti,kw  **#3** 'digital technology'/exp  **#4** 'digital technology':ti,ab,kw OR 'digital technolog*':ab,ti,kw OR 'technolog*, digital':ab,ti,kw OR 'digital electronics':ab,ti,kw OR 'electronics, digital':ab,ti,kw  **#5** 'mobile application'/exp  **#6** 'application*, mobile':ab,ti,kw OR 'mobile app*':ab,ti,kw OR 'app*, mobile':ab,ti,kw OR 'portable software app*':ab,ti,kw OR 'app*, portable software':ab,ti,kw OR 'software app*, portable':ab,ti,kw OR 'smartphone app*':ab,ti,kw OR 'app*, smartphone':ab,ti,kw OR 'portable electronic app*':ab,ti,kw OR 'app*, portable electronic':ab,ti,kw OR 'electronic app*, portable':ab,ti,kw  **#7** 'web-based intervention'/exp  **#8** 'internet based intervention*':ab,ti,kw OR 'intervention*, internet-based':ab,ti,kw OR 'web-based intervention*':ab,ti,kw OR 'intervention*, web-based':ab,ti,kw OR 'online intervention*':ab,ti,kw OR 'intervention*, online':ab,ti,kw OR 'internet intervention*':ab,ti,kw OR 'intervention*, internet':ab,ti,kw  **#9** 'wearable computer'/exp  **#10** 'wearable electronic devices':ab,ti,kw OR 'device, wearable electronic':ab,ti,kw OR 'electronic device, wearable':ab,ti,kw OR 'wearable electronic device':ab,ti,kw OR 'wearable technolog*':ab,ti,kw OR 'technology, wearable':ab,ti,kw OR 'wearable device*':ab,ti,kw OR 'device, wearable':ab,ti,kw OR 'electronic skin':ab,ti,kw  **#11** 'virtual reality'/exp  **#12** 'reality, virtual':ti,ab,kw  **#13** 'virtual medicine':ab,ti,kw OR 'mobile health':ab,ti,kw OR mhealth:ab,ti,kw OR telehealth:ab,ti,kw OR ehealth:ab,ti,kw  **#14** 'telemedicine'/exp  **#15** 'text messaging'/exp  **#16** 'messag*, text':ab,ti,kw OR texting*:ab,ti,kw OR 'short message service':ab,ti,kw OR 'text message*':ab,ti,kw  **#17** 'social media'/exp  **#18** 'social medium':ab,ti,kw OR 'twitter messaging':ab,ti,kw OR 'web 2.0*':ab,ti,kw OR wechat:ab,ti,kw  **#19** 'multimedia'/exp  **#20** game:ab,ti,kw OR gaming:ab,ti,kw OR video:ab,ti,kw  **#21** #1 OR #2 OR #3 OR #4 OR #5 OR #6 OR #7 OR #8 OR #9 OR #10 OR #11 OR #12 OR #13 OR #14 OR #15 OR #16 OR #17 OR #18 OR #19 OR #20  **#22** 'heart failure':ti,ab,kw OR 'cardiac failure':ti,ab,kw OR 'congestive heart failure':ti,ab,kw OR 'chronic heart failure':ti,ab,kw OR 'myocardial failure':ti,ab,kw OR 'heart decompensation':ti,ab,kw OR 'hfref':ti,ab,kw OR 'hfpef':ti,ab,kw  **#23** 'heart failure'/exp  **#24** #22 OR #23  **#25** 'exercise test'/exp  **#26** 'exercise tolerance'/exp  **#27** 'walk test':ti,ab,kw OR '6-minute walk test':ti,ab,kw OR 'six-minute walk test':ti,ab,kw OR '6 minute walk test':ti,ab,kw OR '6-mwt':ti,ab,kw OR '6-min walk test':ti,ab,kw  **#28** 'quality of life'/exp  **#29** 'kccq':ti,ab,kw OR 'kccq-12':ti,ab,kw OR 'kansas city cardiomyopathy questionnaire':ti,ab,kw  **#30** 'minnesota living heart failure questionnaire':ti,ab,kw OR 'mlhfq':ti,ab,kw OR 'chronic heart failure questionnaire':ti,ab,kw OR 'chfq':ti,ab,kw  **#31** 'exercise ability':ti,ab,kw OR 'exercise capacity':ti,ab,kw  **#32** 'quality of life':ti,ab,kw OR 'qol':ti,ab,kw  **#33** 'oxygen consumption'/exp  **#34** 'peak oxygen consumption':ti,ab,kw OR 'peak vo2':ti,ab,kw  **#35** 'cpet':ti,ab,kw  **#36**#25 OR #26 OR #27 OR #28 OR #29 OR #30 OR #31 OR #32 OR #33 OR #34 OR #35  **#37** #21 AND #24 AND #36 | 2106 |
| Cochrane Library | **#1** MeSH descriptor: [Artificial Intelligence] explode all trees  **#2** ((Intelligence, Artificial OR Computational Intelligence OR Intelligence, Computational OR Machine Intelligence OR Computer Reasoning):ti,ab,kw OR (AI (Artificial Intelligence) OR Computer Vision System* OR System*, Computer Vision OR Vision System*, Computer OR Knowledge Acquisition):ti,ab,kw OR (Acquisition, Knowledge (Computer) OR Knowledge Representation*)):ti,ab,kw  **#3** MeSH descriptor: [Digital Technology] explode all trees  **#4** (Digital Technolog* OR Technolog*, Digital OR Digital Electronics OR Electronics, Digital):ti,ab,kw  **#5** MeSH descriptor: [Mobile Applications] explode all trees  **#6** ((Application*, Mobile OR Mobile App* OR App*, Mobile OR Portable Software App* OR App*, Portable Software):ti,ab,kw OR (Software App*, Portable OR Smartphone App* OR App*, Smartphone OR Portable Electronic App* OR App*, Portable Electronic):ti,ab,kw OR (Electronic App*, Portable)):ti,ab,kw  **#7** MeSH descriptor: [Internet-Based Intervention] explode all trees  **#8** ((Internet Based Intervention* OR Intervention*, Internet-Based OR Web-based Intervention* OR Intervention*, Web-based OR Online Intervention*)OR (Intervention*, Online OR Internet Intervention* OR Intervention*, Internet)):ti,ab,kw  **#9** MeSH descriptor: [Wearable Electronic Devices] explode all trees  **#10** (Wearable Device* OR Device, Wearable OR Electronic Skin):ti,ab,kw  **#11** (Device, Wearable Electronic OR Electronic Device, Wearable OR Wearable Electronic Device OR Wearable Technolog* OR Technology, Wearable):ti,ab,kw  **#12** MeSH descriptor: [Virtual Reality] explode all trees  **#13** (Reality, Virtual):ti,ab,kw  **#14** MeSH descriptor: [Telemedicine] explode all trees  **#15** (Virtual Medicine OR Mobile Health OR mHealth OR Telehealth OR eHealth):ti,ab,kw  **#16** MeSH descriptor: [Text Messaging] explode all trees  **#17** (Messag*, Text OR Texting* OR Short Message Service OR Text Message*):ti,ab,kw  **#18** MeSH descriptor: [Social Media] explode all trees  **#19** (Social Medium OR Twitter Messaging OR Web 2.0* OR WeChat):ti,ab,kw  **#20** MeSH descriptor: [Multimedia] explode all trees  **#21** (Game OR gaming OR video):ti,ab,kw  **#22** #1 OR #2 OR #3 OR #4 OR #5 OR #6 OR #7 OR #8 OR #9 OR #10 OR #11 OR #12 OR #13 OR #14 OR #15 OR #16 OR #17 OR #18 OR #19 OR #20 OR #21  **#23** MeSH descriptor: [Heart Failure] explode all trees  **#24** (Heart failure OR cardiac failure OR congestive heart failure OR chronic heart failure OR myocardial failure OR heart decompensation OR HFrEF OR HFpEF):ti,ab,kw  **#25** #23 OR #24  **#26** MeSH descriptor: [Exercise Test] explode all trees  **#27** MeSH descriptor: [Exercise Tolerance] explode all trees  **#28** MeSH descriptor: [Quality of Life] explode all trees  **#29** MeSH descriptor: [Oxygen Consumption] explode all trees  **#30** ("Walk Test" OR "6-minute walk test" OR "six-minute walk test" OR "6 minute walk test" OR "6-MWT" OR "6-min walk test"):ti,ab,kw  **#31** ("KCCQ" OR "KCCQ-12" OR "Kansas City Cardiomyopathy Questionnaire"):ti,ab,kw  **#32** ("minnesota living heart failure questionnaire" OR "MLHFQ" OR "chronic heart failure questionnaire" OR "CHFQ"):ti,ab,kw  **#33** ("accelerometer"):ti,ab,kw  **#34** ("peak oxygen consumption" OR "Peak VO2"):ti,ab,kw  **#35** ("exercise ability" OR "exercise capacity"):ti,ab,kw  **#36** ("cardiopulmonary exercise test" OR "CPET"):ti,ab,kw  **#37** ("quality of life" OR "QoL"):ti,ab,kw  **#38**# 26 OR #27 OR #28 OR #29 OR #30 OR #31 OR #32 OR #33 OR #34 OR #35 OR #36 OR #37  **#39**#22 AND #25 AND #38 | 775 |
| Web of Science | **#1:**TS=("Heart failure" OR "cardiac failure" OR "congestive heart failure" OR "chronic heart failure" OR "myocardial failure" OR "Heart Decompensation" OR "HFrEF" OR "HFpEF")  **#2:**TS=(Game OR gaming OR video) OR TS=(multimedia) OR TS=(Social Media OR Social Medium OR Twitter Messaging OR Web 2.0* OR WeChat) OR TS=(Text Messaging OR Messag*, Text OR Texting* OR Short Message Service OR Text Message*) OR TS=(Telemedicine OR Virtual Medicine OR Mobile Health OR mHealth OR Telehealth OR eHealth) OR TS=(Virtual Reality OR Reality, Virtual) OR TS=(Wearable Electronic Devices OR Device, Wearable Electronic OR Electronic Device, Wearable OR Wearable Electronic Device OR Wearable Technolog* OR Technology, Wearable OR Wearable Device* OR Device, Wearable OR Electronic Skin) OR TS=(Internet-Based Intervention OR Internet Based Intervention* OR Intervention*, Internet-Based OR Web-based Intervention* OR Intervention*, Web-based OR Online Intervention* OR Intervention*, Online OR Internet Intervention* OR Intervention*, Internet) OR TS=(Mobile Applications OR Application*, Mobile OR Mobile App* OR App*, Mobile OR Portable Software App* OR App*, Portable Software OR Software App*, Portable OR Smartphone App* OR App*, Smartphone OR Portable Electronic App* OR App*, Portable Electronic OR Electronic App*, Portable) OR TS=(Digital Technology OR Digital Technolog* OR Technolog*, Digital OR Digital Electronics OR Electronics, Digital) OR TS=(Artificial Intelligence OR Intelligence, Artificial OR Computational Intelligence OR Intelligence, Computational OR Machine Intelligence OR Computer Reasoning OR AI (Artificial Intelligence) OR Computer Vision System* OR System*, Computer Vision OR Vision System*, Computer OR Knowledge Acquisition (Computer) OR Acquisition, Knowledge (Computer) OR Knowledge Representation*)  **#3:**TS=("Walk Test" OR "6-minute walk test" OR "six-minute walk test" OR "6 minute walk test" OR "6-MWT" OR "6-min walk test" OR "KCCQ" OR "KCCQ-12" OR "Kansas City Cardiomyopathy Questionnaire" OR "accelerometer" OR "peak oxygen consumption" OR "cardiopulmonary exercise test" OR "Peak VO2" OR "CPET" OR "Exercise Test" OR "quality of life" OR "minnesota living heart failure questionnaire" OR "MLHFQ" OR "chronic heart failure questionnaire" OR "CHFQ" OR "exercise ability" OR "exercise capacity" OR "Health-Related Quality of Life" OR "QoL" OR "executive function")  **#4:**#1 AND #2 AND #3 | 823 |
| JBI | **1:**("heart failure" or "cardiac failure" or "congestive heart failure" or "chronic heart failure" or "myocardial failure" or "heart decompensation" or "hfref" or "hfpef").ab,kw,ti.  **2:**("game" or "gaming" or "video" or "multimedia" or "social media" or "social medium" or "twitter messaging" or "web 2.0$" or "wechat" or "text messaging" or "messag$, text" or "texting$" or "short message service" or "text message$" or "telemedicine" or "virtual medicine" or "mobile health" or "mhealth" or "telehealth" or "ehealth" or "virtual reality" or "reality, virtual" or "wearable electronic devices" or "device, wearable electronic" or "electronic device, wearable" or "wearable electronic device" or "wearable technolog$" or "technology, wearable" or "wearable device$" or "device, wearable" or "electronic skin" or "internet-based intervention" or "internet based intervention$" or "intervention$, internet-based" or "web-based intervention$" or "intervention$, web-based" or "online intervention$" or "intervention$, online" or "internet intervention$" or "intervention$, internet" or "mobile applications" or "application$, mobile" or "mobile app$" or "app$, mobile" or "portable software app$" or "app$, portable software" or "software app$, portable" or "smartphone app$" or "app$, smartphone" or "portable electronic app$" or "app$, portable electronic" or "electronic app$, portable" or "digital technology" or "digital technolog$" or "technolog$, digital" or "digital electronics" or "electronics, digital" or "artificial intelligence" or "ai" or "computational intelligence" or "machine intelligence" or "computer reasoning" or "computer vision system$" or "computer" or "knowledge representation$").ab,kw,ti.  **3:**("walk test" or "6-minute walk test" or "six-minute walk test" or "6 minute walk test" or "6-mwt" or "6-min walk test" or "kccq" or "kccq-12" or "kansas city cardiomyopathy questionnaire" or "accelerometer" or "peak oxygen consumption" or "cardiopulmonary exercise test" or "peak vo2" or "cpet" or "exercise test" or "quality of life" or "minnesota living heart failure questionnaire" or "mlhfq" or "chronic heart failure questionnaire" or "chfq" or "exercise ability" or "exercise capacity" or "quality of life" or "health-related quality of life" or "qol" or "cognition" or "executive function" or "cognitive" or "memory" or "attention" or "executive function" or "cognitive function$" or "functio$").ab,kw,ti.  **4:**1 and 2 and 3 | 2 |
