# Supplementary material for: Digital Health Technology for Improving Physical Function in Adults With Chronic Heart Failure: Systematic Review and Meta-Analysis of Randomized Controlled Trials
Source: J Med Internet Res. 2026 Aug 13;28:e91820. doi: 10.2196/91820 (PMC13472526; doi:10.2196/91820)
Supplement: Supplementary file 3 [file jmir-v28-e91820-s002.docx]

| **Study (First Author, Year)** | **Exercise Prescription** | **Remote Supervision (Human-in-the-loop)** | **Physiologic / Symptom Monitoring** | **Real-time Feedback / Closed-loop** | **Behavioral Theory / Gamification / Social** | **Final Classification** |
| --- | --- | --- | --- | --- | --- | --- |
| **Piotrowicz et al (2020) [38]**  (TELEREH-HF) | Yes | Yes (Tele-ECG & team) | Yes | Yes | Yes | **Hybrid** |
| **Szalewska et al (2021) [37]** (TELEREH-HF) | Yes | Yes (Tele-ECG & team) | Yes | Yes | Yes | **Hybrid** |
| **Piotrowicz et al (2022) [29]** (TELEREH-HF) | Yes | Yes (Tele-ECG & team) | Yes | Yes | Yes (Tele-psychology) | **Hybrid** |
| **Langlo et al (2022) [31]** (IT IS HOPE) | Yes | Yes (Real-time video) | Yes | Yes | No | **Hybrid** |
| **Lundgren et al (2023) [28]** (IT IS HOPE) | Yes | Yes (Real-time video) | Yes | Yes | No | **Hybrid** |
| **Lundgren et al (2025) [23]**  **(IT IS HOPE)** | Yes | Yes (Real-time video) | Yes | Yes | No | **Hybrid** |
| **Lödding et al (2024) [24] (HITS)** | Yes | Yes (Study staff review) | Yes | Yes | No | **Hybrid** |
| **Song et al (2024) [25]** (H2H+O2O) | Yes | Yes (Specialist nurse) | Yes | Yes | Yes (Reward points) | **Hybrid** |
| **Wang and Zhu (2023) [27]** (WeChat) | Yes | Yes (Doctor/Nurse <6h) | Yes | Yes | Yes (Patient circle) | **Hybrid** |
| **Nagatomi et al (2022) [32]** | Yes | Yes (CR team weekly) | Yes (Fitbit) | Yes | No | **Hybrid** |
| **Peng et al (2018) [39]** | Yes | Yes (WeChat/QQ team) | Yes (HR monitor) | Yes | No | **Hybrid** |
| **Hwang et al (2017) [40]** | Yes | Yes (Real-time video) | Yes | Yes | Yes (Group support) | **Hybrid** |
| **Smolis-Bąk et al (2015) [41]** | Yes | Yes (ECG monitoring center) | Yes | Yes | No | **Hybrid** |
| **Radhakrishnan et al (2021) [33]** | No | No (Automated) | Yes (Sensors) | No (Game loop only) | Yes (Fogg model, game) | **Standalone** |
| **Felker et al (2022) [30]** (TARGET-HF-DM) | No | No (Automated texts) | Yes (Step counter) | No | No | **Standalone** |
| **Nolan et al (2021) [34]** (CHF-CePPORT) | No | No (Automated) | Yes | No | Yes (CBT, MI) | **Standalone** |
| **Saleh et al (2022) [26]** | No | No | Yes | No | No | **Standalone** |
| **Jaarsma et al (2021) [36]** (HF-Wii) | Yes (30m) | No (Only motivation call) | No | No | Yes (Game) | **Standalone** |
| **Clays et al (2021) [35]** (HeartMan) | Yes | No (Automated DSS) | Yes (Wristband) | No | Yes (Mindfulness, CBT) | **Standalone** |
| **Blomqvist et al (2025) [25]** (HEALTHY) | No | No (Automated) | Yes | No | Yes | **Standalone** |

References

22. Blomqvist A, Bäck M, Klompstra L, Strömberg A, Jaarsma T. Testing the recruitment frequency, implementation fidelity, and feasibility of outcomes of the Heart Failure Activity Coach Study (HEALTHY): pilot randomized controlled trial. JMIR Form Res. Jan 8, 2025;9:e62910. [doi: 10.2196/62910] [Medline: 39778202]

23. Lundgren KM, Langlo KAR, Salvesen Ø, et al. Exercise-based telerehabilitation for heart failure patients declining outpatient rehabilitation—a randomized controlled trial. Med Sci Sports Exerc. Mar 1, 2025;57(3):449-460. [doi: 10.1249/MSS.0000000000003590] [Medline: 39501477]

24. Lödding P, Beyer S, Pökel C, et al. Adherence to long-term telemonitoring-supported physical activity in patients with chronic heart failure. Sci Rep. Sep 26, 2024;14(1):22037. [doi: 10.1038/s41598-024-70371-0] [Medline: 39327450]

25. Song YJ, Xu JQ, Chen X, et al. Formulation and application of the “hospital-to-home + online-to-offline” treatment scheme in caring for and supporting vulnerable patients with chronic heart failure. Technol Health Care. 2024;32(1):191-200. [doi: 10.3233/THC-220693] [Medline: 37393446]

26. Saleh ZT, Elshatarat RA, Elhefnawy KA, et al. Effect of a home-based mobile health app intervention on physical activity levels in patients with heart failure: a randomized controlled trial. J Cardiovasc Nurs. 2023;38(2):128-139. [doi: 10.1097/JCN.0000000000000911] [Medline: 35389920]

27. Wang S, Zhu MY. Impact of WeChat-based “hospital-home” integrated health education on exercise minded patients with chronic heart failure. Rev Int Med Cienc Act Fis Deporte. 2023;23(89):170-183. [doi: 10.15366/rimcafd2022.89.012]

28. Lundgren KM, Langlo KAR, Salvesen Ø, et al. Feasibility of telerehabilitation for heart failure patients inaccessible for outpatient rehabilitation. ESC Heart Fail. Aug 2023;10(4):2406-2417. [doi: 10.1002/ehf2.14405] [Medline: 37221704]

29. Piotrowicz E, Mierzyńska A, Jaworska I, et al. Relationship between physical capacity and depression in heart failure patients undergoing hybrid comprehensive telerehabilitation vs. usual care: subanalysis from the TELEREH-HF randomized clinical trial. Eur J Cardiovasc Nur. Aug 29, 2022;21(6):568-577. [doi: 10.1093/eurjcn/zvab125]

30. Felker GM, Sharma A, Mentz RJ, et al. A randomized controlled trial of mobile health intervention in patients with heart failure and diabetes. J Card Fail. Nov 2022;28(11):1575-1583. [doi: 10.1016/j.cardfail.2022.07.048] [Medline: 35882260]

31. Langlo KAR, Lundgren KM, Zanaboni P, et al. Cardiorenal syndrome and the association with fitness: data from a telerehabilitation randomized clinical trial. ESC Heart Fail. Aug 2022;9(4):2215-2224. [doi: 10.1002/ehf2.13985] [Medline: 35615893]

32. Nagatomi Y, Ide T, Higuchi T, et al. Home-based cardiac rehabilitation using information and communication technology for heart failure patients with frailty. ESC Heart Fail. Aug 2022;9(4):2407-2418. [doi: 10.1002/ehf2.13934] [Medline: 35534907]

33. Radhakrishnan K, Julien C, Baranowski T, et al. Feasibility of a sensor-controlled digital game for heart failure self-management: randomized controlled trial. JMIR Serious Games. Nov 8, 2021;9(4):e29044. [doi: 10.2196/29044] [Medline: 34747701]

34. Nolan RP, Ross HJ, Farkouh ME, et al. Automated e-counseling for chronic heart failure: CHF-CePPORT trial. Circ Heart Fail. Jan 2021;14(1):e007073. [doi: 10.1161/CIRCHEARTFAILURE.120.007073] [Medline: 33464959]

35. Clays E, Puddu PE, Luštrek M, et al. Proof-of-concept trial results of the HeartMan mobile personal health system for self-management in congestive heart failure. Sci Rep. Mar 11, 2021;11(1):5663. [doi: 10.1038/s41598-021-84920-4] [Medline: 33707523]

36. Jaarsma T, Klompstra L, Ben Gal T, et al. Effects of exergaming on exercise capacity in patients with heart failure: results of an international multicentre randomized controlled trial. Eur J Heart Fail. Jan 2021;23(1):114-124. [doi: 10.1002/ejhf.1754] [Medline: 32167657]

37. Szalewska D, Główczyńska R, Piotrowicz R, et al. An aetiology-based subanalysis of the Telerehabilitation in Heart Failure Patients (TELEREH-HF) trial. ESC Heart Fail. Apr 2021;8(2):1263-1273. [doi: 10.1002/ehf2.13189] [Medline: 33527740]

38. Piotrowicz E, Pencina MJ, Opolski G, et al. Effects of a 9-week hybrid comprehensive telerehabilitation program on long-term outcomes in patients with heart failure: the Telerehabilitation in Heart Failure Patients (TELEREH-HF) randomized clinical trial. JAMA Cardiol. Mar 1, 2020;5(3):300-308. [doi: 10.1001/jamacardio.2019.5006] [Medline: 31734701]

39. Peng X, Su Y, Hu Z, et al. Home-based telehealth exercise training program in Chinese patients with heart failure: a randomized controlled trial. Medicine (Baltimore). Aug 2018;97(35):e12069. [doi: 10.1097/MD.0000000000012069] [Medline: 30170422]

40. Hwang R, Bruning J, Morris NR, Mandrusiak A, Russell T. Home-based telerehabilitation is not inferior to a centre-based program in patients with chronic heart failure: a randomised trial. J Physiother. Apr 2017;63(2):101-107. [doi: 10.1016/j.jphys.2017.02.017] [Medline: 28336297]

41. Smolis-Bąk E, Dąbrowski R, Piotrowicz E, et al. Hospital-based and telemonitoring guided home-based training programs: effects on exercise tolerance and quality of life in patients with heart failure (NYHA class III) and cardiac resynchronization therapy. A randomized, prospective observation. Int J Cardiol. Nov 15, 2015;199(442-7):442-447. [doi: 10.1016/j.ijcard.2015.07.041] [Medline: 26276068]
