# Supplementary material for: Digital Health Technology for Improving Physical Function in Adults With Chronic Heart Failure: Systematic Review and Meta-Analysis of Randomized Controlled Trials
Source: J Med Internet Res. 2026 Aug 13;28:e91820. doi: 10.2196/91820 (PMC13472526; doi:10.2196/91820)
Supplement: Supplementary file 4 [file jmir-v28-e91820-s003.docx]

**Six-minute walk test (6MWT)**

| **Certainty assessment** | | | | | | | **№ of patients** | | **Effect** | | **Certainty** | **Importance** |
| --- | --- | --- | --- | --- | --- | --- | --- | --- | --- | --- | --- | --- |
| **№ of studies** | **Study design** | **Risk of bias** | **Inconsistency** | **Indirectness** | **Imprecision** | **Other considerations** | **[intervention]** | **[comparison]** | **Relative (95% CI)** | **Absolute (95% CI)** |  |  |
| 10 | randomised trials | serious^a^ | not serious^b^ | not serious | not serious | none^c^ | 1067 | 1050 | - | MD **18.74 m higher** (9.31 higher to 28.17 higher) | ⨁⨁⨁◯ Moderate^a,b,c^ |  |

**CI:** confidence interval; **MD:** mean difference

#### Explanations

a. Most studies are open label, and the 6-minute walk test is a semi-objective indicator that is easily influenced by patients' subjective effort and motivation (Performance/Detection bias). Some studies, such as Nagatomi, have high risks. Down-graded one level.

b. Although I² = 71% (substantial heterogeneity), the majority of the point estimates in the studies are on the right side of the zero line (tending to be valid), with consistent directions, only differing in the magnitude of the effects.

c. Visual inspection of the funnel plot did not reveal obvious asymmetry, suggesting a low risk of publication bias.

**Peak VO2 (pVO2)**

| **Certainty assessment** | | | | | | | **№ of patients** | | **Effect** | | **Certainty** |
| --- | --- | --- | --- | --- | --- | --- | --- | --- | --- | --- | --- |
| **№ of studies** | **Study design** | **Risk of bias** | **Inconsistency** | **Indirectness** | **Imprecision** | **Other considerations** | **[intervention]** | **[comparison]** | **Relative (95% CI)** | **Absolute (95% CI)** |  |
| 3 | randomised trials | serious^a^ | not serious | not serious | not serious^b^ | none^c^ | 479 | 478 | - | MD **0.84 mL/kg/min higher** (0.59 higher to 1.09 higher) | ⨁⨁⨁◯ Moderate^a,b,c^ |

**CI:** confidence interval; **MD:** mean difference

#### Explanations

a. Because of non-blind design (Open label). Although Peak VO₂ is a physiological indicator with less subjective influence, considering the high-risk evaluation of some studies, it is conservatively downgraded by one level.

b. The sample size is robust (N=957), and the 95% CI [0.59, 1.09] is narrow and significant.

c. Not assessed. The number of studies was insufficient to construct a funnel plot.

**Objectively Measured Daily Step Count**

| **Certainty assessment** | | | | | | | **№ of patients** | | **Effect** | | **Certainty** | **Importance** |
| --- | --- | --- | --- | --- | --- | --- | --- | --- | --- | --- | --- | --- |
| **№ of studies** | **Study design** | **Risk of bias** | **Inconsistency** | **Indirectness** | **Imprecision** | **Other considerations** | **[intervention]** | **[comparison]** | **Relative (95% CI)** | **Absolute (95% CI)** |  |  |
| **New outcome** | | | | | | | | | | | | |
| 3 | randomised trials | serious^a^ | very serious^b^ | not serious | serious^c^ | none | 148 | 153 | - | MD **1.51 thousand steps higher** (1.78 lower to 4.79 higher) | ⨁◯◯◯ Very low^a,b,c,d^ |  |

**CI:** confidence interval; **MD:** mean difference

#### Explanations

a. According to the RoB diagram, the included studies (such as Felker, Saleh) have "Some concerns" and due to the nature of the intervention (wearing devices), it is not possible to blind the participants (Performance bias).

b. The heterogeneity is extremely high(I² = 99%). The research results are completely inconsistent in direction (Saleh significantly increases, Blomqvist decreases), and the confidence intervals have almost no overlap. Cannot be explained by a single effect quantity.

c. 95% CI [-1.78, 4.79] crossed the invalid line (0) and the range was extremely wide (from decrease to significant increase), with a relatively small sample size (N=301).

d.Due to the small number of included studies (n < 10), funnel plot asymmetry could not be assessed; however, the presence of studies with null or negative effects suggests publication bias is unlikely.

**MLHFQ**

| **Certainty assessment** | | | | | | | **№ of patients** | | **Effect** | | **Certainty** | **Importance** |
| --- | --- | --- | --- | --- | --- | --- | --- | --- | --- | --- | --- | --- |
| **№ of studies** | **Study design** | **Risk of bias** | **Inconsistency** | **Indirectness** | **Imprecision** | **Other considerations** | **[intervention]** | **[comparison]** | **Relative (95% CI)** | **Absolute (95% CI)** |  |  |
| **New outcome** | | | | | | | | | | | | |
| 4 | randomised trials | serious^a^ | not serious | not serious | not serious | none^b^ | 169 | 172 | - | MD **5.63 lower** (8.55 lower to 2.71 lower) | ⨁⨁⨁◯ Moderate^a,b^ |  |

**CI:** confidence interval; **MD:** mean difference

#### Explanations

a. Quality of life is a subjective reported outcome (PROs) that is highly susceptible to placebo effects (Performance/Detection bias) in non blinded trials. And it includes high-risk studies (such as the presence of red lights in Wang 2023 and Lundgren 2024).

b.Not assessment. The number of studies was insufficient to construct a funnel plot.

**KCCQ**

| **Certainty assessment** | | | | | | | **№ of patients** | | **Effect** | | **Certainty** | **Importance** |
| --- | --- | --- | --- | --- | --- | --- | --- | --- | --- | --- | --- | --- |
| **№ of studies** | **Study design** | **Risk of bias** | **Inconsistency** | **Indirectness** | **Imprecision** | **Other considerations** | **[intervention]** | **[comparison]** | **Relative (95% CI)** | **Absolute (95% CI)** |  |  |
| **New outcome** | | | | | | | | | | | | |
| 3 | randomised trials | serious^a^ | serious^b^ | not serious | serious^c^ | none^d^ | 108 | 112 | - | MD **0.22 lower** (7.68 lower to 7.24 higher) | ⨁◯◯◯ Very low^a,b,c,d^ |  |

**CI:** confidence interval; **MD:** mean difference

#### Explanations

a. Subjective outcome, non blind design. Nagatomi is considered high-risk.

b. I² = 73%，Inconsistent direction of results (Felker significantly positive, Radhakrishnan negative)

c. The 95% CI [-7.68, 7.24] is extremely broad, crossing the invalid line and encompassing both the clinical extremes of "deterioration" and "improvement", with highly uncertain results.

d.Due to the small number of included studies (n < 10), funnel plot asymmetry could not be assessed; however, the presence of studies with null or negative effects suggests publication bias is unlikely.
