# Supplementary material for: Digital Health Technology for Improving Physical Function in Adults With Chronic Heart Failure: Systematic Review and Meta-Analysis of Randomized Controlled Trials
Source: J Med Internet Res. 2026 Aug 13;28:e91820. doi: 10.2196/91820 (PMC13472526; doi:10.2196/91820)
Supplement: Supplementary file 5 [file jmir-v28-e91820-s004.docx]

| **Study (First Author, Year)** | **Device Type / Brand** | **Wear Location** | **Minimum Wear-Time / Data Validation Criteria** | **Baseline & Post-Intervention Consistency** |
| --- | --- | --- | --- | --- |
| Felker et al (2022) [30] | Withings Go® commercial tracking device | Wrist-worn | Baseline:≥2days of data in Week 1 within a physiologic range of 200–20,000 steps/day. Month 3: ≥21$ days (≥25% of expected days) from days 8–91. | Yes. Steps tracked continuously using identical cloud-based software at both time points. |
| Saleh et al (2023) [26] | Samsung mobile health application | Pant pocket | Monitored continuously over a 2-week pre-intervention baseline and an 8-week intervention period; verified via weekly participant screenshots. | Yes. Evaluated using the exact same built-in smartphone application parameters across all phases. |
| Blomqvist et al (2025) [22] | ActiGraph GT9X research-grade accelerometer | Hip-worn | Instructed to wear for 7 consecutive days in Weeks 1 and 12. Valid data required ≥4days with a minimum wear time of 540 minutes/day. Non-wear time defined as ≥60 consecutive minutes of zero counts. | Yes. Standardized ActiLife software and identical filtering algorithms applied consistently. |

References

22. Blomqvist A, Bäck M, Klompstra L, Strömberg A, Jaarsma T. Testing the recruitment frequency, implementation fidelity, and feasibility of outcomes of the Heart Failure Activity Coach Study (HEALTHY): pilot randomized controlled trial. JMIR Form Res. Jan 8, 2025;9:e62910. [doi: 10.2196/62910] [Medline: 39778202]

26. Saleh ZT, Elshatarat RA, Elhefnawy KA, et al. Effect of a home-based mobile health app intervention on physical activity levels in patients with heart failure: a randomized controlled trial. J Cardiovasc Nurs. 2023;38(2):128-139. [doi: 10.1097/JCN.0000000000000911] [Medline: 35389920]

30. Felker GM, Sharma A, Mentz RJ, et al. A randomized controlled trial of mobile health intervention in patients with heart failure and diabetes. J Card Fail. Nov 2022;28(11):1575-1583. [doi: 10.1016/j.cardfail.2022.07.048] [Medline: 35882260]
